# The correlation between the trajectory of plasma atherosclerosis-inducing index in the examination population and the risk of developing metabolic dysfunction-associated steatotic liver disease

**DOI:** 10.3389/fendo.2026.1708671

**Published:** 2026-03-31

**Authors:** Yongxin Li, Changying Zhao, Jun Wang, Tao Shi

**Affiliations:** 1 Department of Cardiovascular Surgery, The First Affiliated Hospital of Xi’an Jiaotong University, Xi’an, Shaanxi, China; 2 Department of Health Sciences, The First Affiliated Hospital of Xi’an Jiaotong University, Xi’an, Shaanxi, China

**Keywords:** atherogenic index of plasma, group-based trajectory modeling, longitudinal study, metabolic dysfunction-associated steatotic liver disease, risk prediction

## Abstract

**Background:**

Metabolic Dysfunction-Associated Steatotic Liver Disease (MASLD) is a prevalent metabolic disorder with rising global incidence and substantial clinical implications. The atherogenic index of plasma (AIP), a novel marker of lipid metabolism, has shown potential in predicting metabolic diseases, but its longitudinal association with MASLD remains unclear.

**Methods:**

This longitudinal study included 1,894 participants from the health examination database of the First Affiliated Hospital of Xi’an Jiaotong University (2020–2024). Group-based trajectory modeling (GBTM) identified three AIP trajectory patterns: low-stable, moderate-stable, and high-stable. Cox proportional hazards models were used to assess the association between AIP trajectories and incident MASLD, adjusting for demographic, lifestyle, and metabolic factors. Subgroup and sensitivity analyses were conducted to assess effect modification and robustness.

**Results:**

Three AIP trajectory patterns were identified: low-stable (33.7%), moderate-stable (49.1%), and high-stable (17.3%). Over a median follow-up of 2.97 years, the high-stable group exhibited a 2.23-fold increased risk of incident MASLD (adjusted HR = 2.23, 95% CI: 1.51–3.30; P < 0.001) compared with the low-stable group, even after adjusting for metabolic mediators including BMI and fasting glucose. The moderate-stable group also showed an increased risk (HR = 1.42, 95% CI: 1.02–1.98; P = 0.038). Subgroup analyses demonstrated consistent associations, with more pronounced effects among non-diabetic and non-hypertensive individuals. Sensitivity analyses further confirmed the robustness of these findings.

**Conclusions:**

Sustained high AIP levels are independently associated with an increased risk of MASLD. AIP trajectory monitoring may offer a valuable tool for early identification and targeted prevention of MASLD.

## 1 Introduction

In recent years, metabolic dysfunction-associated steatotic liver disease (MASLD) has emerged as a significant global health challenge. MASLD has been estimated to affect 30% of the adult population worldwide, with its prevalence increasing from 22% to 37% from 1991 to 2019 (1). Observations in 2016–2019 showed an alarming trend and a further increase in the percentage of patients with liver steatosis to 30.5% (2). MASLD is a group of heterogeneous diseases characterized by the presence of diffuse fatty liver on imaging technique or histological features of significant macrovesicular steatosis (3). Moreover, MASLD is associated with an increased risk of cardiovascular disease, higher all-cause mortality, and a significantly higher incidence of type 2 diabetes and adverse pregnancy outcomes, such as a 2.1-fold increase in preterm birth and a 3.4-fold increase in macrosomia (4–6). By 2040, over half the adult population is forecasted to have MASLD. The largest increases are expected to occur in women, smokers, and those without metabolic syndrome (7).This trend urgently calls for the development of more accurate prediction tools.

The Atherogenic Index of Plasma (AIP) has been recognized as a reliable surrogate indicator for evaluating dyslipidemia and insulin resistance (8). Unlike traditional lipid tests, AIP can reflect the dynamic balance between atherogenic lipoproteins and protective lipoproteins (9). Notably, the latest meta-analysis shows that AIP performs better in predicting MASLD, with an area under the curve (AUC) of 0.764, which is significantly better than single lipid parameters (8). All in all, as an emerging biomarker, AIP has also been widely used in the prediction of cardiovascular diseases, pre-diabetic lesions, and MASLD (10, 11).

However, existing studies exhibit notable knowledge gaps. Most adopt a static design, failing to capture the dynamic relationship between changes in AIP and MASLD progression. Consequently, the underlying mechanisms and long-term effects remain inadequately explored. For example, a recent study by Yan Chen et al. found a significant dose-response relationship between elevated AIP and the prevalence of MASLD, but the long-term effects and mechanisms remain to be explored (12). Population-based trajectory modeling has been successfully used to analyze cardiovascular metabolic indicators. Recently, Sun et al. demonstrated that AIP trajectories are potent predictors for the onset of type 2 diabetes in a large longitudinal health-screened cohort (13). However, the application of this framework to studying the link between AIP and new-onset MASLD remains underexplored (14, 15).

This study explores the link between AIP and MASLD by analyzing longitudinal AIP changes and their correlation with FLD development using hospital health check-up data from 2020 to 2024. Population trajectory modeling identified unique AIP trends, highlighting its potential in predicting FLD. The findings provide important evidence linking obesity to liver disease risk, offering a valuable basis for improving early screening strategies and guiding more precise, personalized interventions.

## 2 Methods

### 2.1 Study design and participants

**Figure 1:**
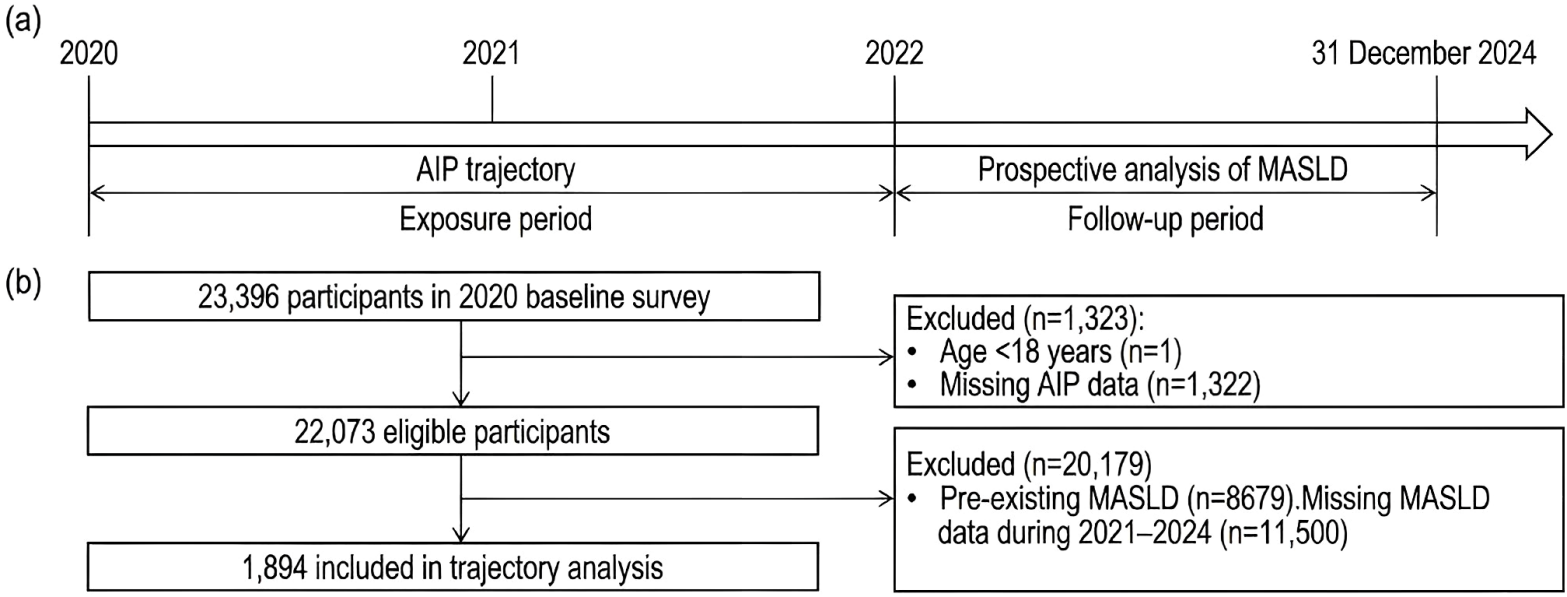
Study design and participant selection flowchart. AIP, atherogenic index of plasma; MASLD, metabolic dysfunction-associated steatotic liver disease. **(a)** Timeline diagram showing the exposure period (2020–2022) and follow-up period (until 31 December 2024). **(b)** Flowchart showing participant selection, with 1,894 participants ultimately included in the trajectory analysis.

This longitudinal cohort study used data from the health examination database of the First Affiliated Hospital of Xi’an Jiaotong University, covering the period from 2020 to 2024. The outcome event was defined as incident MASLD, diagnosed based on imaging-confirmed or biopsy-proven hepatic steatosis in conjunction with metabolic dysfunction, according to international consensus criteria. Cases were identified using the relevant International Classification of Diseases (ICD-10) codes. Participants were included if they had at least three valid AIP measurements during the exposure period (2020–2022), ensuring sufficient data for trajectory modeling. Exclusion criteria were: (1) age <18 years as of 2020; (2) missing baseline AIP data in 2020; (3) a diagnosis of MASLD prior to or during the exposure period (2020–2022); and (4) incomplete follow-up data for MASLD outcomes during the follow-up period (2023–2024). The final analytic cohort comprised 1,894 participants who met all inclusion criteria with no exclusions (**Figure 1**). This cohort provided data for both AIP trajectory modeling (exposure period: 2020–2022) and the prospective analysis of incident MASLD (follow-up period: 2023–2024).

This study complied with the Declaration of Helsinki and was approved by the Ethics Committee of the First Affiliated Hospital of Xi’an Jiaotong University (Approval No: XJTU1AF2025LSYY-679). Due to its retrospective design using anonymized health examination data, the Ethics Committee granted a waiver for individual informed consent.

### 2.2 Diagnostic criteria for MASLD

MASLD was defined according to the international consensus criteria as the presence of imaging-confirmed hepatic steatosis together with at least one metabolic risk factor (16). In this cohort, hepatic steatosis was primarily identified by abdominal ultrasonography during routine health examinations.

Incident MASLD was defined as ultrasound-confirmed hepatic steatosis together with at least one metabolic risk factor, including overweight/obesity (BMI ≥23 kg/m²), hyperglycemia (fasting plasma glucose ≥5.6 mmol/L or physician-diagnosed diabetes), hypertension (SBP ≥130 mmHg or DBP ≥85 mmHg or physician-diagnosed hypertension), or dyslipidemia (triglycerides ≥1.7 mmol/L or reduced HDL cholesterol <1.0 mmol/L in men or <1.3 mmol/L in women).

### 2.3 Data collection

Data collection encompassed age, gender, body mass index (BMI), systolic blood pressure (SBP), high-density lipoproteincholesterol (HDL-C), uric acid (UA), fasting blood glucose (FBG), total cholesterol (TC), and estimated glomerular filtration rate (eGFR).

After fasting for at least 8h, each participant had blood drawn for analysis, which was subsequently evaluated using an automatic biochemical analyzer. The researchers evaluated the lifestyle characteristics of the participants using a standardized questionnaire, which included information on smoking, drinking, physical exercise, history of hypertension, history of diabetes, and history of cardiovascular diseases. To address missing data, we employed multiple imputation under the missing-at-random assumption.

Trained and experienced medical staff collected anthropometric and clinical data using standardized protocols: The atherogenic index of plasma was calculated as the base-10 logarithm of the ratio of triglycerides (TG) to high-density lipoprotein cholesterol (17).


$$AIP\;=\;\log_{10}\left(\frac{TG}{HDL\;-\;C}\right)$$

### 2.4 Statistical analysis

We performed group-based trajectory modeling (GBTM) using the PROC TRAJ procedure in SAS 9.4 to identify distinct AIP trajectories during 2020-2022 (18, 19). Specifically, we used baseline AIP measurements from 2020 and follow-up measurements from 2021 and 2022 as trajectory points to model the temporal patterns of AIP changes, while utilizing the 2023-2024 follow-up data to assess the occurrence of endpoint events. Model selection was based on the following criteria (20): (1) smaller bayesian information criterion (BIC) values indicating better fit; (2) each trajectory class containing ≥5% of participants; (3) mean posterior probabilities of class membership >70% for all trajectories; (4) odds of correct classification >5 for each trajectory group; and (5) clear visual distinction between trajectories with clinically interpretable patterns. The optimal number of trajectories was determined by comparing 2- to 4-group solutions. The 3-group model (BIC = 964.67) was selected for its superior classification accuracy (Min. APP = 0.886; Min. OCC = 7.75) and adherence to the minimum 5% group size requirement. The 4-group solution was rejected as its smallest group comprised only 3.3% of the cohort (**Supplementary Table 1**). we ultimately identified three distinct AIP trajectory patterns: low-stable, moderate-stable, and high-stable groups. These trajectory models not only met all statistical criteria but also demonstrated good clinical relevance and interpretability. The final model was selected after evaluating multiple functional forms (linear, quadratic, and cubic for each trajectory group to ensure optimal fit to the observed data patterns (21).

Demographic and clinical characteristics were compared across AIP trajectory groups using ANOVA for normally distributed continuous variables, Kruskal-Wallis test for non-normally distributed variables, and x^2^ tests for categorical variables. We employed cox proportional hazards regression models to examine the association between AIP trajectories and incident MASLD, with sequential adjustment in four models: Model 0 (unadjusted); Model 1 (adjusted for age and sex); Model 2 (additional adjustment for current smoking, current alcohol, exercise, history of diabetes, history of hypertension and history of cardiovascular disease) and Model 3 (further adjustment for BMI, SBP, UA, FBG, eGFR, Hb and TC), eGFR and Hb were adjusted in Model 3 as potential confounders related to metabolic health, given that both impaired kidney function (reflected by low eGFR) and anemia (indicated by low Hb levels) are frequently associated with worsening metabolic disorders and could influence the development of MASLD. Additionally, we assessed model stability by calculating the Events-Per-Variable (EPV), which reflects the number of incident cases of MASLD per covariate. In our fully adjusted Model 3, we included 13 covariates, with a total of 172 incident MASLD cases, resulting in an EPV of approximately 13.2 (172 events/13 covariates). This EPV exceeds the commonly recommended threshold of 10. To clarify the causal assumptions underlying our adjustment strategy, a directed acyclic graph (DAG) was constructed to depict the hypothesized relationships among AIP trajectories, demographic and lifestyle factors, metabolic indicators, and MASLD risk (**Supplementary Figure 2**). The DAG was used to differentiate confounders from potential mediators and to guide the sequential adjustment models. The proportional hazards (PH) assumption was verified using Schoenfeld residual tests and visual inspection of log-log survival plots, which confirmed no significant violation for the AIP trajectory groups (P = 0.831) or the global model (P = 0.248). The corresponding Schoenfeld residual plots are presented in **Supplementary Figure 1**.

To explore potential effect modification, we conducted stratified subgroup analyses based on sex (male vs. female), age (<47 vs. ≥47 years), current drinking status (yes vs. no), regular exercise (yes vs. no), history of diabetes (yes vs. no), history of hypertension (yes vs. no), BMI (<23.56 vs. ≥23.56 kg/m²), SBP (SBP <120 vs. ≥120 mmHg), FBG (FBG <4.67 vs. ≥4.67 mmol/L), eGFR (eGFR <108.91 vs. ≥108.91 mL/min/1.73 m²), Hb (Hb <138 vs. ≥138 g/L), and TC (TC <4.56 vs. ≥4.56 mmol/L).

For each subgroup, cox proportional hazards models were fitted to estimate the association between AIP trajectory patterns and MASLD risk, using the low-stable group as the reference. Interaction terms were added to assess statistical significance of effect modification. In addition, sensitivity analyses were performed to assess the robustness of the findings. We repeated the main models across several restricted populations, including individuals aged ≥40 years, those aged 30–70 years, and participants without a history of chronic diseases (those without diabetes, hypertension, or cardiovascular disease), using four progressive models with increasing covariate adjustments. All statistical analyses were performed using R (version 4.5.0), and a two-sided α level of 0.05 was used to define statistical significance.

## 3 Results

### 3.1 Baseline characteristics

**Figure 2:**
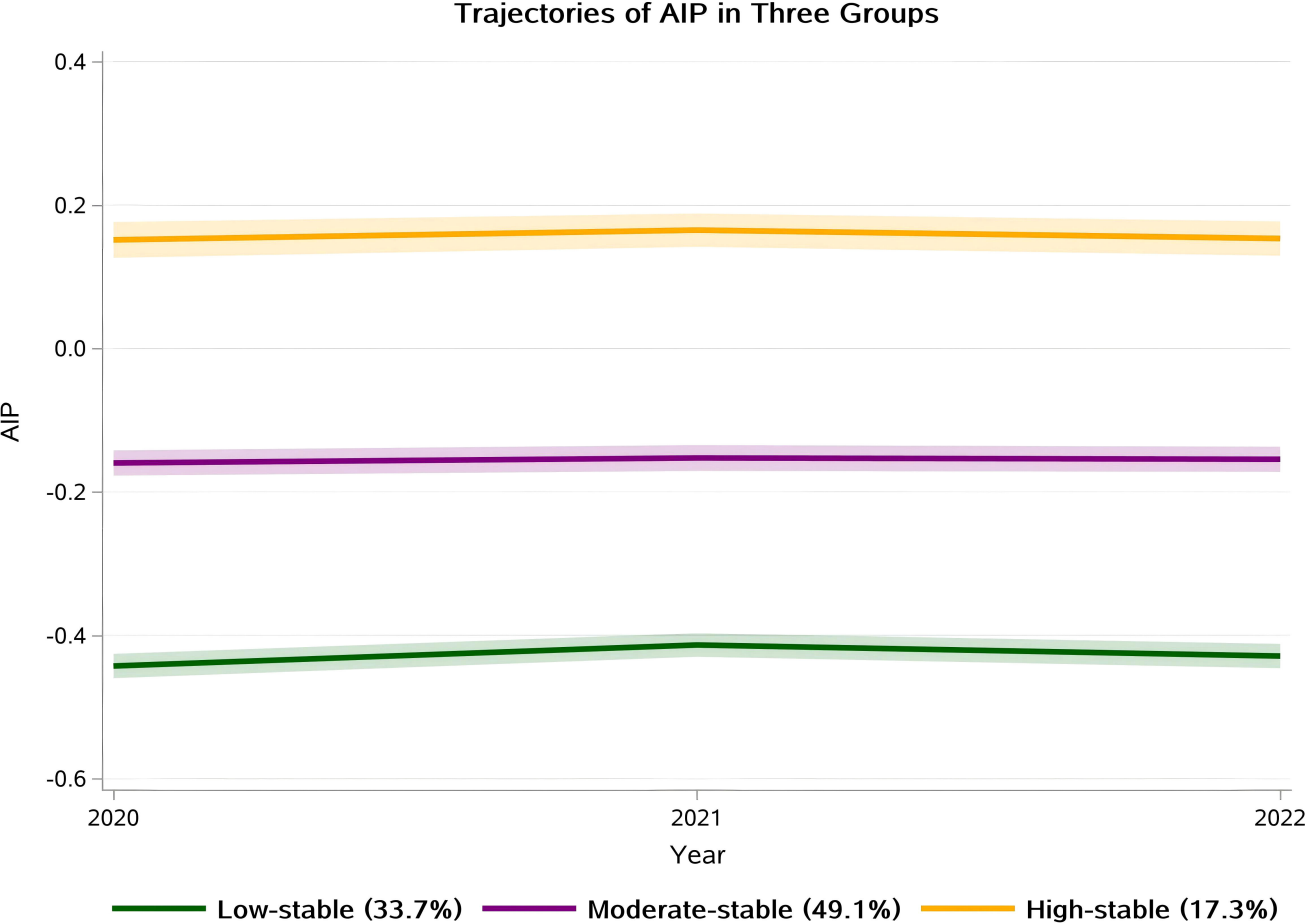
Trajectories of MASLD development by AIP Groups. Group 1: Low-stable group; Group 2: Moderate-stable group; Group 3: High-stable group.

Over the 3-year period, 1,894 participants exhibited three stable AIP trajectories (**Figure 2**): low-stable (n=628, 33.7%; mean AIP: -0.44, slope: 0.05, P = 0.002), moderate-stable (n=950, 49.1%; mean: -0.16, slope: 0.01, P = 0.418), and high-stable (n=316, 17.3%; mean: 0.15, slope: 0.03, P = 0.271). The actual mean AIP values for each year and the estimated slopes for each trajectory group are detailed in **Supplementary Table 2**. Baseline characteristics of the study population according to the three AIP trajectory groups are presented in **Table 1**. Participants in the high-stable group were older (mean age: 52.05 ± 14.90 years) and had a higher proportion of males (48.4%) compared to those in the moderate-stable and low-stable groups (28.9% and 14.8%, respectively; P < 0.001). The prevalence of current smoking and drinking increased progressively from the low-stable to high-stable groups (P < 0.001 and P = 0.006, respectively). Individuals in the high-stable group also had higher frequencies of hypertension (30.1%), diabetes (5.7%), and cardiovascular disease (5.4%) compared to the other groups.

**Table 1:** Baseline characteristics of the study participants according to the trajectories of the AIP.

Characteristics	Overall	AIP trajectories			P value
		Low‐stable(n=628)	Moderate‐stable(n=950)	High‐stable(n=316)	
Age, (y)	48.5 ± 14.3	45.28 ± 13.39	49.35 ± 14.23	52.05 ± 14.90	<0.001
Male, n(%)	521(27.5)	93(14.8)	275(28.9)	153(48.4)	<0.001
Current smoking, n(%)	160(8.4)	33(5.3)	79(8.3)	48(15.2)	<0.001
Current drinking, n(%)	387(20.4)	103(16.4)	207(21.8)	77(24.4)	0.006
Exercise, n (%)	1166(61.6)	387(61.6)	594(62.5)	185(58.5)	0.451
History of hypertension, n(%)	372(19.6)	75(11.9)	202(21.3)	95(30.1)	<0.001
History of diabetes history, n(%)	64(3.4)	15(2.4)	31(3.3)	18(5.7)	0.028
History of CVD, n(%)	73(3.9)	11(1.8)	45(4.7)	17(5.4)	0.003
BMI (kg/m²)	21.8 ± 2.3	21.00 ± 2.18	22.09 ± 2.25	22.79 ± 2.16	<0.001
SBP (mmHg)	122.3 ± 17.1	118.82 ± 15.64	122.53 ± 16.85	128.62 ± 18.66	<0.001
UA(µmol/L)	283.6 ± 70.7	258.78 ± 58.65	287.07 ± 68.88	322.32 ± 78.39	<0.001
FBG(mmol/L)	4.8 ± 0.9	4.66 ± 0.69	4.81 ± 0.92	5.02 ± 1.29	<0.001
eGFR (mL/min/1.73m²)	107.3 ± 14.6	110.74 ± 12.86	106.61 ± 14.52	102.32 ± 16.45	<0.001
Hb(g/L)	138.5 ± 15.1	135.10 ± 13.01	138.83 ± 15.79	144.29 ± 14.75	<0.001
TC(mmol/L)	4.6 ± 0.9	4.50 ± 0.82	4.62 ± 0.89	4.78 ± 0.98	<0.001

Values are presented as mean ± standard deviation (for continuous variables) or number (percentage) for categorical variables. P values were derived using ANOVA for continuous variables and chi-square test for categorical variables across AIP trajectory groups. AIP, Atherogenic Index of Plasma; CVD, Cardiovascular Disease; BMI, Body Mass Index; SBP, Systolic Blood Pressure; UA, Uric Acid; FBG, Fasting Blood Glucose; eGFR, Estimated Glomerular Filtration Rate; Hb, Hemoglobin; TC, Total Cholesterol.

Significant differences were observed across groups in metabolic parameters. Participants in the high-stable group exhibited higher levels of BMI (22.79 ± 2.16 kg/m²), SBP (128.62 ± 18.66 mmHg), UA (322.32 ± 78.39 µmol/L), and FBG (5.02 ± 1.29 mmol/L), along with lower eGFR (102.32 ± 16.45 mL/min/1.73m²), compared with those in the low-stable and moderate-stable groups (all P < 0.001). Similarly, Hb and TC levels were significantly elevated in the high-stable group. No significant difference was observed in regular exercise habits across the three groups (P = 0.451).

### 3.2 Association between AIP trajectories and risk of incident MASLD

**Figure 3:**
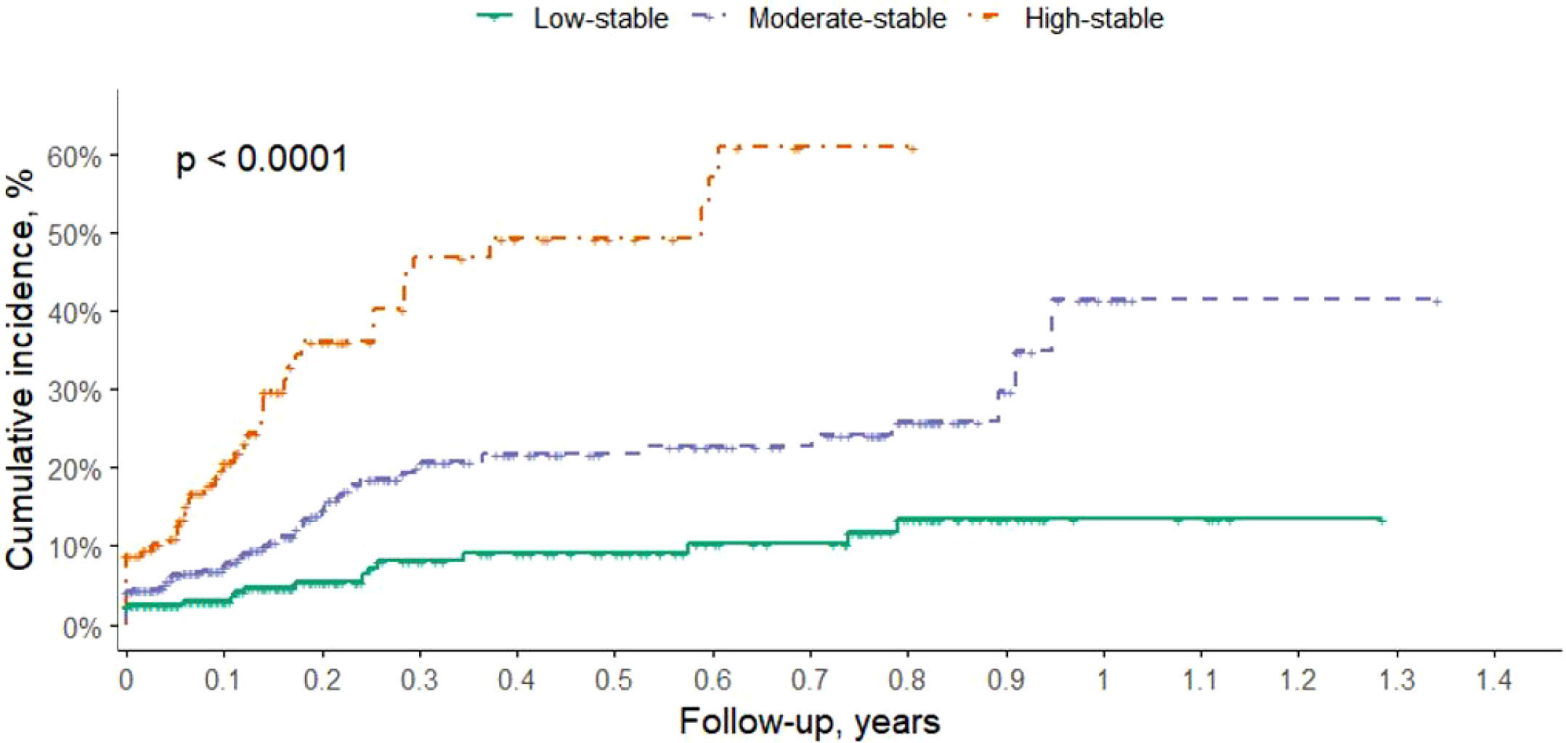
Cumulative Incidence of MASLD by AIP Trajectory Groups. Kaplan–Meier curves showing the cumulative incidence of fatty liver across three distinct AIP trajectory groups: low-stable, moderate-stable, and high-stable. The log-rank test indicated a statistically significant difference among the groups (p < 0.0001). The shaded areas represent 95% confidence intervals. The numbers at risk below the x-axis represent the number of participants remaining under observation at each time point within each group. AIP, atherogenic index of plasma.

During the follow-up period, 172 incident cases (9.1%) of MASLD were identified among the 1,894 participants. The number of events in the low-stable, moderate-stable, and high-stable AIP trajectory groups were 27 (4.3%), 81 (8.5%), and 64 (20.3%), respectively. The cumulative incidence varied substantially across the three groups as illustrated in **Figure 3**. Participants in the high-stable AIP trajectory group showed a markedly increased cumulative incidence of fatty liver during follow-up, reaching over 50%, compared with approximately 35% in the moderate-stable group and less than 15% in the low-stable group. The log-rank test indicated a statistically significant difference among the trajectory groups (P < 0.0001), suggesting a strong association between sustained high AIP levels and elevated MASLD risk.

Multivariable Cox regression models further confirmed this association (**Table 2**). In the unadjusted model (Model 0), compared to the low-stable group, the moderate-stable and high-stable groups had significantly higher risks of incident fatty liver, with hazard ratios (HRs) of 2.32 (95% CI: 1.50–3.59) and 5.76 (95% CI: 3.66–9.05), respectively (P_trend < 0.001). These associations remained robust after adjusting for age and sex (Model 1), as well as lifestyle factors and comorbidities (Model 2). In the fully adjusted model (Model 3), which additionally accounted for metabolic parameters including BMI, SBP, UA, FBG, eGFR, Hb and TC, the HRs were attenuated but remained statistically significant (HR = 1.42 for moderate-stable and 2.23 for high-stable; P_trend = 0.002). To further characterize the shape of the dose-response relationships for key continuous metabolic variables, restricted cubic spline (RCS) analyses were performed (**Supplementary Figure 3**). After adjusting for AIP trajectory groups and other confounders, significant nonlinear associations with MASLD risk were observed for FBG (P for nonlinearity = 0.0256) and Hb (P for nonlinearity = 0.1125), with both variables showing statistically significant overall associations (FBG: P = 0.0066; Hb: P = 0.0136). BMI demonstrated a strong overall association with MASLD risk (P < 0.0001), which was predominantly linear in nature (P for nonlinearity = 0.0986). Age, eGFR, and TC did not show statistically significant overall associations in this analysis. Detailed P-values for overall and nonlinear associations are provided in **Supplementary Table 3**.

**Table 2:** Association between MASLD trajectory groups and clinical outcomes.

Characteristics	AIP trajectory, HR (95%)			P trend
	Low‐stable	Moderate‐stable	High‐stable	
Events (%)	27 (4.3)	81 (8.5)	64 (20.3)	
Model 0	1.00 (Ref)	2.32 (1.50–3.59)	5.76 (3.66–9.05)	< 0.001
Model 1	1.00 (Ref)	1.87 (1.20–2.92)	3.66 (2.28–5.88)	< 0.001
Model 2	1.00 (Ref)	1.84 (1.18–2.87)	3.53 (2.19–5.69)	< 0.001
Model 3	1.00 (Ref)	1.42 (0.91–2.23)	2.23 (1.35–3.69)	0.002

Model 0: Unadjusted; Model 1: Adjusted for age and sex; Model 2: Model 1 + current smoking, current alcohol, exercise, history of diabetes, history of hypertension and history of cardiovascular disease; Model 3: Model 2 + body mass index, systolic blood pressure, uric acid, fasting blood glucose, estimated glomerular filtration rate, hemoglobin and total cholesterol.

### 3.3 Incremental predictive value of AIP trajectories

To establish clinical utility, we benchmarked the AIP trajectory-enhanced model against established non-invasive liver scores. When added to the baseline clinical model (C-index: 0.700), the AIP trajectory yielded the highest predictive accuracy (C-index: 0.723), outperforming both FIB-4 (C-index: 0.714) and NFS (C-index: 0.691). These findings highlight that longitudinal AIP monitoring offers superior incremental value for identifying individuals at high risk of developing MASLD compared to traditional fibrosis-oriented tools.

### 3.4 Subgroup and sensitivity analysis

Subgroup analyses demonstrated that the association between AIP trajectories and fatty liver risk was generally consistent across most strata, including sex, age, drinking status, physical activity, BMI, and metabolic profiles (**Table 3**). However, significant interaction effects were observed for diabetes (P interaction = 0.004), hypertension (P interaction < 0.001), and systolic blood pressure (P interaction = 0.034), indicating effect modification by these factors. Specifically, the association was stronger in participants without diabetes or hypertension, as well as those with lower SBP (<120mmHg). No other subgroups showed significant interactions.

**Table 3:** Subgroup analysis of AIP trajectories for MASLD.

Subgroups	N	Low-stable	Moderate-stable	High-stable	P for trend	P for interaction
		HR (Ref)	HR (95% CI)	HR (95% CI)		
**Sex**						0.098
Female	1373	1.00 (ref)	2.23 (1.25–4.00)	5.99 (3.13–11.45)	< 0.001	
Male	521	1.00 (ref)	1.33 (0.68–2.58)	2.26 (1.16–4.40)	0.004	
Age (years)						0.075
< 47	927	1.00 (ref)	2.10 (1.15–3.81)	7.75 (4.20–14.30)	< 0.001	
≥ 47	967	1.00 (ref)	2.24 (1.16–4.32)	3.89 (1.97–7.69)	< 0.001	
Current drinking						0.55
No	1507	1.00 (ref)	2.00 (1.21–3.32)	4.70 (2.74–8.04)	< 0.001	
Yes	387	1.00 (ref)	3.27 (1.35–7.95)	7.63 (3.12–18.63)	< 0.001	
Exercise						0.406
No	728	1.00 (ref)	1.89 (0.96–3.70)	6.27 (3.17–12.41)	< 0.001	
Yes	1166	1.00 (ref)	2.66 (1.49–4.72)	5.49 (2.99–10.07)	< 0.001	
Diabetes						**< 0.001**
No	1830	1.00 (ref)	2.65 (1.66–4.22)	6.97 (4.31–11.29)	< 0.001	
Yes	64	1.00 (ref)	0.34 (0.08–1.44)	0.24 (0.04–1.41)	0.107	
Hypertension						**0.004**
No	1522	1.00 (ref)	3.16 (1.84–5.40)	7.86 (4.49–13.74)	< 0.001	
Yes	372	1.00 (ref)	0.68 (0.32–1.47)	1.54 (0.71–3.35)	0.165	
BMI (kg/m^2)^						**0.014**
< 23.56	1495	1.00 (ref)	1.90 (1.10–3.29)	6.24 (3.52–11.07)	< 0.001	
≥ 23.56	480	1.00 (ref)	1.91 (0.84–4.34)	2.31 (1.00–5.35)	0.056	
DBP (mmHg)						0.623
< 73	966	1.00 (ref)	2.65 (1.40–5.03)	4.96 (2.35–10.46)	< 0.001	
≥ 73	991	1.00 (ref)	1.81 (0.98–3.36)	4.38 (2.36–8.13)	< 0.001	
FBG (mmol/L)						0.481
< 4.67	942	1.00 (ref)	2.40 (1.22–4.71)	7.07 (3.55–14.05)	< 0.001	
≥ 4.67	952	1.00 (ref)	1.87 (1.05–3.32)	4.08 (2.23–7.44)	< 0.001	
eGFR (mL/min)						0.193
< 108.91	946	1.00 (ref)	2.41 (1.22–4.79)	4.43 (2.20–8.92)	< 0.001	
≥ 108.91	948	1.00 (ref)	2.03 (1.14–3.63)	7.09 (3.84–13.08)	< 0.001	
Hb (g/L)						0.474
< 138	898	1.00 (ref)	1.86 (0.89–3.91)	6.14 (2.66–14.17)	< 0.001	
≥ 138	1001	1.00 (ref)	2.10 (1.21–3.65)	4.15 (2.36–7.28)	< 0.001	
TC (mmol/L)						0.24
< 4.56	937	1.00 (ref)	2.19 (1.20–4.00)	7.15 (3.90–13.12)	< 0.001	
**≥** 4.56	957	1.00 (ref)	2.34 (1.24–4.42)	4.36 (2.21–8.59)	< 0.001	

AIP, Atherogenic Index of Plasma; BMI, Body Mass Index; CI, Confidence Interval; eGFR, estimated Glomerular Filtration Rate; FBG, Fasting Blood Glucose; Hb, Hemoglobin; HR, Hazard Ratio; SBP, Systolic Blood Pressure; TC, Total Cholesterol.

The low-stable AIP trajectory served as the reference group. P_trend values indicate dose-response effects across trajectories. P_interaction values assess effect modification by subgroups. Significant interactions (P<0.05) suggest stronger associations in metabolically healthy subgroups. Bold values indicate interaction P < 0.05.

Sensitivity analyses confirmed the robustness of our findings (**Table 4**). The positive association between AIP trajectories and MASLD risk persisted across subgroups defined by age. A lag-time analysis excluding events in the first three months yielded consistent results (high-stable vs. low-stable: HR = 2.06, 95% CI: 1.23–3.44, P = 0.006), with a significant dose-response trend (P_trend < 0.001).

**Table 4:** Sensitivity analysis of association between AIP trajectory and risk of MASLD.

	Model	Low-stable	Moderate-stable	High-stable	P trend
Age ≥ 40					
	Model 0	1.00 (Ref)	1.80 (1.07–3.02)	4.37 (2.55–7.48)	<0.001
	Model 1	1.00 (Ref)	1.63 (0.97–2.75)	3.42 (1.97–5.94)	0.004
	Model 2	1.00 (Ref)	1.56 (0.92–2.63)	3.22 (1.84–5.61)	<0.001
	Model 3	1.00 (Ref)	1.27 (0.75–2.16)	2.35 (1.32–4.19)	0.004
Age 30–70					
	Model 0	1.00 (Ref)	2.43 (1.47–4.01)	6.68 (3.98–11.23)	<0.001
	Model 1	1.00 (Ref)	2.02 (1.21–3.36)	4.41 (2.54–7.66)	<0.001
	Model 2	1.00 (Ref)	2.02 (1.21–3.37)	4.25 (2.45–7.38)	<0.001
	Model 3	1.00 (Ref)	1.53 (0.91–2.56)	2.65 (1.51–4.66)	<0.001
Excluding events within first 3 months					
	Model 0	1.00 (Ref)	2.32 (1.50–3.59)	5.76 (3.66–9.05)	<0.001
	Model 1	1.00 (Ref)	1.87 (1.20–2.92)	3.66 (2.28–5.88)	<0.001
	Model 2	1.00 (Ref)	1.84 (1.18–2.87)	3.53 (2.19–5.69)	<0.001
	Model 3	1.00 (Ref)	1.41 (0.90–2.23)	2.06 (1.23–3.44)	0.004

Reference group: Low-stable AIP trajectory. Values are hazard ratios (HRs) with 95% confidence intervals (CI), estimated from Cox proportional hazards models. Model 0: Unadjusted; Model 1: Adjusted for age and sex; Model 2: Model 1 + smoking, alcohol, exercise, diabetes, hypertension, cardiovascular disease; Model 3: Model 2 + BMI, diastolic BP, uric acid, fasting glucose, eGFR, hemoglobin, total cholesterol.

## 4 Discussion

This longitudinal study investigated the association between AIP trajectories and incident MASLD in a hospital-based cohort. Using GBTM, we identified three distinct AIP patterns—low-stable, moderate-stable, and high-stable. Our findings demonstrated that individuals with persistently high AIP levels had a significantly elevated risk of developing MASLD during follow-up, independent of traditional metabolic risk factors. Our study found that a high-stable AIP trajectory was associated with a 2.23-fold increased risk of MASLD.

The moderate-stable AIP group showed a non-significant association with MASLD risk in Model 3 (HR = 1.42, 95% CI: 0.91–2.23), likely due to metabolic confounders like BMI, blood pressure, and glucose levels. Notably, the association between the high-stable AIP trajectory and MASLD risk remained significant even after adjustment for metabolic factors in Model 3, suggesting that the predictive effect of AIP trajectories cannot be fully explained by these intermediate metabolic pathways. Once these factors were adjusted for, the relationship between AIP and MASLD risk weakened, suggesting that metabolic abnormalities in this group played a larger role. This aligns with studies showing that metabolic syndrome components, such as obesity and insulin resistance, are key contributors to MASLD and may mask the independent effect of AIP on liver disease progression (22). In contrast, the high-stable AIP group maintained a robust risk (HR = 2.23, 95% CI: 1.35–3.69) even after full adjustment, indicating that persistently high AIP may independently contribute to fatty liver risk. This supports findings that atherogenic dyslipidemia, indicated by high AIP, is a distinct factor in MASLD development, independent of traditional metabolic factors. We hypothesize that lipid-lowering therapy (e.g., statins), which effectively reduces AIP levels, may have attenuated the association in the moderate-stable group. The absence of medication data limits our ability to quantify this bias, warranting cautious interpretation of the null finding in this subgroup (23, 24).

Mechanistically, AIP reflects an imbalance between atherogenic and protective lipoproteins, promoting hepatic lipid accumulation through: Increased free fatty acid flux from adipose tissue to the liver due to elevated TG-rich lipoprotein breakdown (27); Impaired β-oxidation caused by HDL-C deficiency, which reduces PPARα-mediated fatty acid oxidation (28); Hepatic insulin resistance triggered by lipid intermediates (e.g., diacylglycerols and ceramides) derived from TG metabolism (29). The stronger association in non-diabetic and non-hypertensive subgroups suggests that AIP may capture early metabolic dysfunction before overt diabetes or hypertension develops (30). This aligns with studies showing AIP correlates with visceral adiposity and subclinical inflammation (elevated hs-CRP and IL-6), which precede glucose dysregulation but directly promote hepatic steatosis through Kupffer cell activation and *de novo* lipogenesis.

Importantly, to offer a comprehensive view of the main co-morbidity, which is cardiovascular disease, the relationship between AIP and the progression of metabolic diseases like MASLD and atherosclerosis is rooted in the complex interplay of lipid dynamics and systemic inflammation. Indeed, AIP serves as a potent proxy for the presence of small, dense low-density lipoprotein (sdLDL) particles, which are highly prone to oxidation. In the context of MASLD, an elevated AIP reflects the metabolic dysregulation and hepatic insulin resistance that drive fat accumulation in the liver, often preceding more severe histological changes (8). As these lipid abnormalities persist, the high concentration of pro-atherogenic particles reflected by a high AIP facilitates their infiltration into the arterial intima. Here, they undergo oxidative modification and trigger the inflammatory cascade characteristic of atherosclerosis. Clinical evidence indicates that an elevated AIP initiates structural thickening of the carotid intima; as the AIP value increases, there is a proportional increase in carotid intima-media thickness (CIMT), making it a more accurate predictor of vascular remodeling than traditional lipid profiles (31). In MASLD, hepatic lipid dysregulation directly accelerates CIMT thickening, linking liver dysfunction to early structural arterial damage. Carotid Doppler ultrasonography thus serves as a vital non-invasive tool for identifying subclinical atherosclerosis in these patients, independent of traditional risk factors (32).

Subgroup analyses revealed that the association between AIP trajectories and MASLD risk was significantly modified by diabetes and hypertension (both PP for interaction < 0.001). As shown in **Table 3**, the positive association was pronounced and statistically significant among participants without diabetes (HR for high-stable group: 6.97, 95% CI: 4.31–11.29) and without hypertension (HR: 7.86, 95% CI: 4.49–13.74). In stark contrast, the association was completely attenuated in participants with prevalent diabetes (HR: 0.24, 95% CI: 0.04–1.41) or hypertension (HR: 1.54, 95% CI: 0.71–3.35) at baseline. A similar pattern of effect modification was observed for BMI (P for interaction = 0.014), where the association was stronger in leaner individuals (BMI < 23.56 kg/m²). The observed effect modification by diabetes and hypertension suggests that in metabolically healthy individuals, atherogenic dyslipidemia (high AIP) represents a predominant risk driver, whereas in those with established metabolic disease, insulin resistance and systemic inflammation may overshadow the contribution of lipid fractions. This aligns with the ‘multiple hits’ hypothesis of MASLD pathogenesis (25, 26, 33). These findings highlight that the predictive value of longitudinal AIP trajectories for MASLD is not uniform but is profoundly influenced by an individual’s underlying metabolic status. The robust association in healthier subgroups underscores the potential utility of AIP as an early warning signal for MASLD risk stratification in primary prevention settings.

Strengths of this study include its longitudinal design, rigorous trajectory modeling, and comprehensive adjustment for confounders. For example, Chun et al. identified increasing vs. decreasing AIP trajectories over 5-7 years using GBTM in the KoGES cohort, showing significantly higher CVD risk in the increasing group (34). Similarly, Li et al., in the 12-year ELSA cohort, robustly adjusted for confounders and found the high-stable AIP trajectory conferred 33 % greater CVD risk (35).These findings highlight the robustness, clinical relevance, and generalizability of GBTM-based AIP trajectory analysis.

However, several important limitations should be considered. First, key confounders—particularly lipid-modifying medications (e.g., statins, fibrates), dietary habits, and genetic predisposition—were not systematically documented. As AIP is highly sensitive to pharmacological modification, the absence of medication data may limit interpretation of subgroup-specific findings, such as the attenuated association observed in participants with diabetes. Second, the hospital-based design of the cohort may limit the generalizability of our findings to the wider population, although sensitivity analyses conducted on healthier subgroups partially addressed this concern. Third, the short follow-up (2023–2024) may have primarily captured early-stage or previously unrecognized steatosis rather than exclusively true new-onset disease. This potential detection bias suggests that our findings are best positioned to support longitudinal AIP monitoring as a practical tool for near-term clinical screening and early risk stratification, rather than long-term disease progression or lifetime risk prediction. Fourth, while rigorous sensitivity and lag-time analyses support our findings’ internal validity, the lack of an external validation cohort limits generalizability. These results provide a foundation that requires confirmation in diverse settings.

## 5 Conclusions

This study identified three distinct AIP trajectory patterns and demonstrated that individuals with persistently elevated AIP levels faced a significantly higher risk of developing MASLD. The association remained robust after comprehensive adjustment for potential confounders and was consistently observed across key subgroups. These findings highlight the clinical utility of longitudinal AIP monitoring for early risk identification. For the high-stable group, ‘targeted prevention’ should include intensive lifestyle interventions, aggressive lipid management, and increased frequency of metabolic screening and liver imaging.

## Data Availability

The raw data supporting the conclusions of this article will be made available by the authors, without undue reservation.
