# Supplementary material for: The correlation between the trajectory of plasma atherosclerosis-inducing index in the examination population and the risk of developing metabolic dysfunction-associated steatotic liver disease
Source: Front Endocrinol (Lausanne). 2026 Mar 31;17:1708671. doi: 10.3389/fendo.2026.1708671 (PMC13076150; doi:10.3389/fendo.2026.1708671)

**Supplementary Document**

**The correlation between the trajectory of plasma atherosclerosis-inducing index in the examination population and the risk of developing metabolic dysfunction-associated steatotic liver disease**

Supplementary Table S1. Model Fit and Selection Diagnostics for AIP Trajectories

| **No. of Groups** | **BIC (N=1894)** | **Group Proportions (%)** | **Smallest Group Size (%)** | **Min. APP** | **Min. OCC** |
| --- | --- | --- | --- | --- | --- |
| 2-group | 621.54 | 61.5%, 38.5% | 0.385 | 0.615 | 1.6 |
| 3-group | 964.67 | 33.7%, 49.1%, 17.3% | 0.173 | 0.886 | 7.75 |
| 4-group | 1127.64 | 24.4%, 45.8%, 26.5%, 3.3% | 0.033 | N/A* | N/A* |

*APP: Average Posterior Probability (Threshold > 0.70); OCC: Odds of Correct Classification (Threshold > 5.0).

Supplementary Table S2. Actual mean AIP values and estimated slopes for the three trajectory groups

| Trajectory Group | 2020  (Mean ± SD) | 2021  (Mean ± SD) | 2022  (Mean ± SD) | Estimated Slope  (P)* |
| --- | --- | --- | --- | --- |
| Low-stable (n=628) | -0.45 ± 0.14 | -0.42 ± 0.14 | -0.44 ± 0.14 | 0.05 (P=0.002) |
| Moderate-stable (n=950) | -0.16 ± 0.14 | -0.15 ± 0.15 | -0.15 ± 0.15 | 0.01 (P=0.418) |
| High-stable (n=316) | 0.16 ± 0.20 | 0.18 ± 0.17 | 0.17 ± 0.19 | 0.03 (P=0.271) |

*Estimated slopes represent the linear term coefficients from the SAS PROC TRAJ output.

Supplementary Table S3. Results of Restricted Cubic Spline (RCS) Analysis for Continuous Modifiers

| Variable | Overall P-value | Non-linear P-value |
| --- | --- | --- |
| Fasting Blood Glucose (mmol/L) | 0.0066 | 0.0256 |
| BMI (kg/m2) | <0.0001 | 0.0986 |
| Hemoglobin (g/L) | 0.0136 | 0.1125 |
| Age (years) | 0.098 | 0.354 |
| eGFR (mL/min/1.73m2) | 0.0831 | 0.1744 |
| Total Cholesterol (mmol/L) | 0.7454 | 0.8757 |

Note: Models were adjusted for AIP trajectory groups, sex, smoking, drinking, exercise, diabetes, and hypertension. The non-linear $P$-value was derived from the Wald test.

Supplementary Figure S1. Testing the Proportional Hazards Assumption via Schoenfeld Residuals.


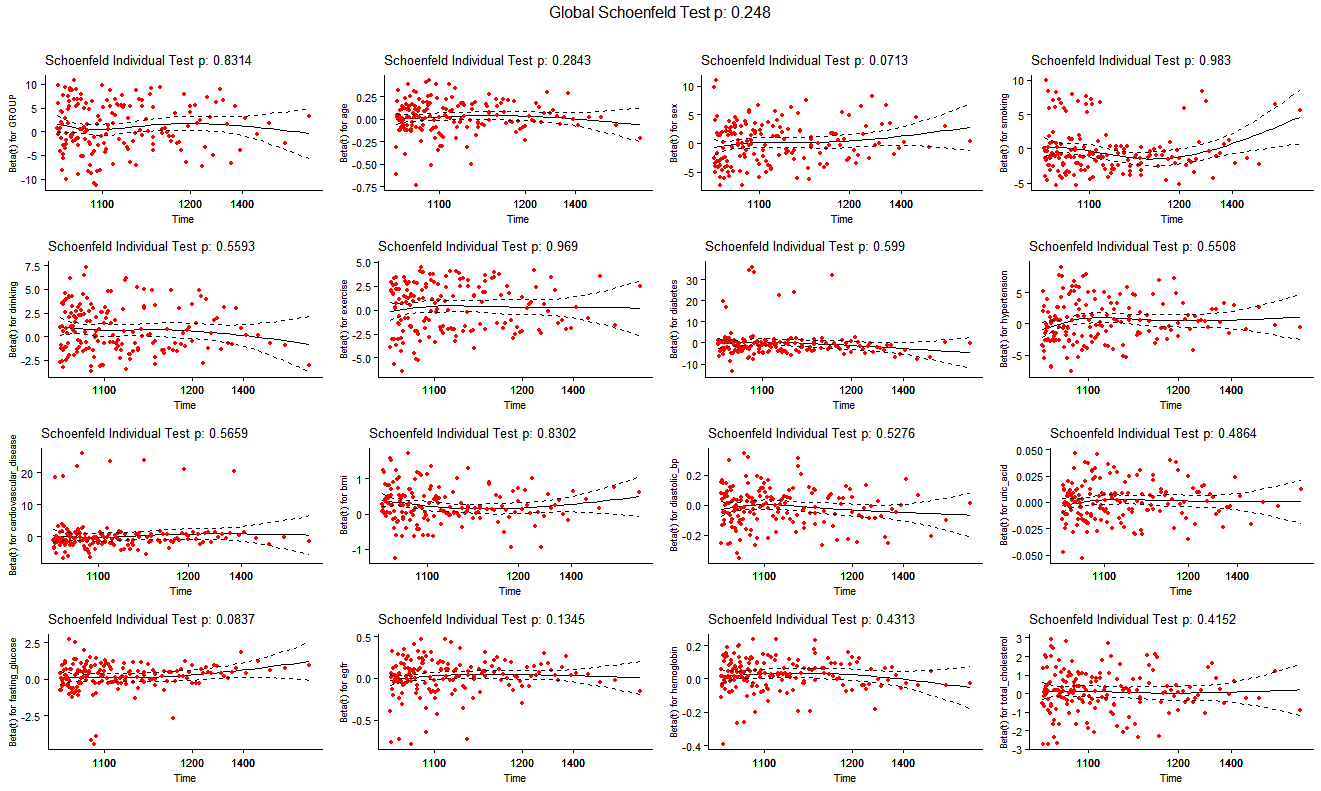


Supplementary Figure S2. Directed Acyclic Graph (DAG) for AIP Trajectories and MASLD.


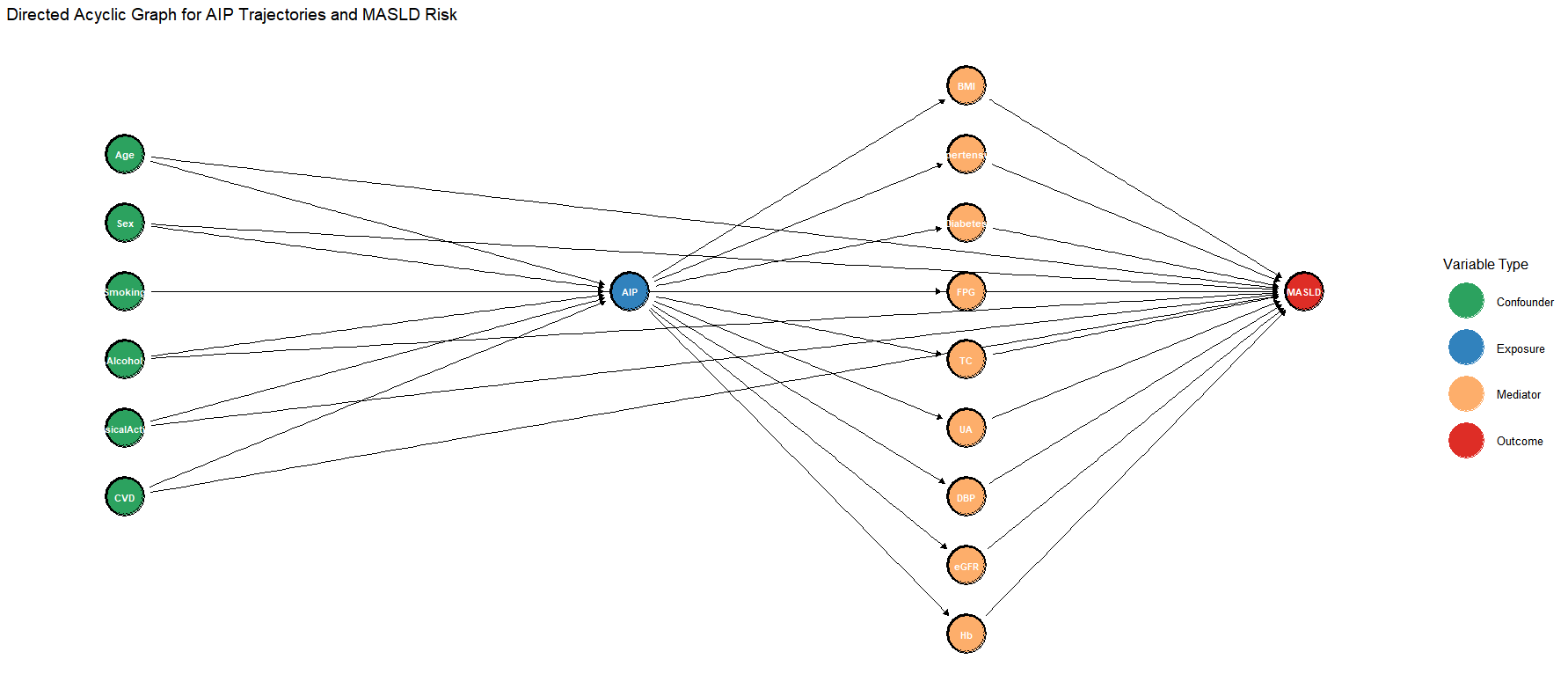


Directed acyclic graph illustrating the hypothesized causal relationships between AIP trajectories and the risk of metabolic dysfunction–associated steatotic liver disease (MASLD). Green nodes represent confounders, the blue node represents the exposure (AIP trajectory), orange nodes represent potential mediators, and the red node represents the outcome (MASLD).

Supplementary Figure S3. Systematic RCS Analysis: Al Continuous Variables vs. MASLD Risk


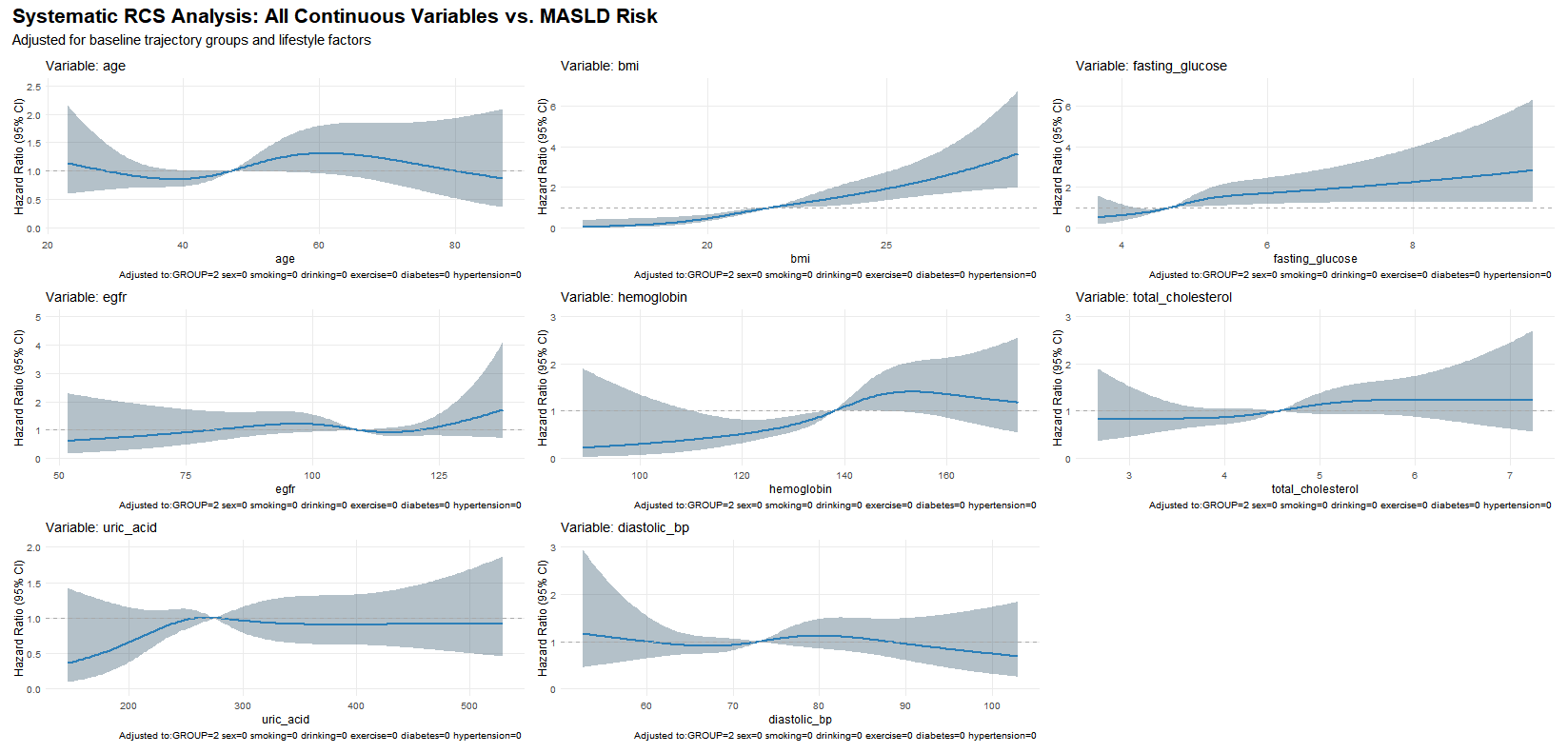

Supplement: Supplementary file 1 [file DataSheet1.docx]
